# Design of new Mott multiferroics via complete charge transfer: promising candidates for bulk photovoltaics

**DOI:** 10.1038/s41598-017-06396-5

**Published:** 2017-07-21

**Authors:** Hanghui Chen, Andrew Millis

**Affiliations:** 1 NYU-ECNU Institute of Physics, NYU Shanghai, Shanghai, 200062 China; 2 Department of Physics, New York University, New York, NY 10002 USA; 3 Department of Physics, Columbia University, New York, NY 10027 USA

## Abstract

Optimal materials to induce bulk photovoltaic effects should lack inversion symmetry and have an optical gap matching the energies of visible radiation. Ferroelectric perovskite oxides such as BaTiO_3_ and PbTiO_3_ exhibit substantial polarization and stability, but have the disadvantage of excessively large band gaps. We use both density functional theory and dynamical mean field theory calculations to design a new class of Mott multiferroics–double perovskite oxides *A*_2_VFeO_6_ (*A* = Ba, Pb, etc). While neither perovskite *A*VO_3_ nor *A*FeO_3_ is ferroelectric, in the double perovskite *A*_2_VFeO_6_ a ‘complete’ charge transfer from V to Fe leads to a non-bulk-like charge configuration–an empty V-*d* shell and a half-filled Fe-*d* shell, giving rise to a polarization comparable to that of ferroelectric *A*TiO_3_. Different from nonmagnetic *A*TiO_3_, the new double perovskite oxides have an antiferromagnetic ground state and around room temperatures, are paramagnetic Mott insulators. Most importantly, the V *d* ^0^ state significantly reduces the band gap of *A*_2_VFeO_6_, making it smaller than that of *A*TiO_3_ and BiFeO_3_ and rendering the new multiferroics a promising candidate to induce bulk photovoltaic effects.

## Introduction

The lack of inversion symmetry caused by ferroelectric ordering in certain transition metal oxides can separate electrons and holes generated by photo-excitation, making these materials promising candidates for photovoltaic devices^1–4^. However, many known ferroelectric perovskite oxides including BaTiO_3_ and PbTiO_3_ have very large band gaps (~3–5 eV)^5^, significantly limiting their absorption efficiency in the visible frequency range. The large band gap is intrinsic: it is set by the energy difference between the Ti-*d* and O-*p* levels, which is large because Ti and O have substantially different electronegativity. Intensive research in perovskite oxides has focused on reducing band gaps while maintaining ferroelectric polarization. One approach is to replace a fraction of transition metal ions with a different cation, with one transition metal species driving ferroelectricity and the other providing lower energy states that reduce the band gap^6–11^. Using this approach, band gap reductions of ~1 eV have been attained^10^ and a high power conversion efficiency has been experimentally achieved in Bi_2_FeCrO_6_^11^. In another method, a class of layered double perovskite oxides *AA*′*BB*′O_6_ has been theoretically proposed, in which a large in-plane polarization is found via nominal *d* ^0^ filling on the *B*-site, *A*-site cations bearing lone-pair electrons, and *A*′ ≠ *A* size mismatch; the band gap is controlled by *B*/*B*′ electronegativity difference^12^.

In this work, we propose a simple design scheme. We introduce a new class of double perovskite oxides *A*_2_VFeO_6_ where *A* is a divalent cation (*A* = Ba, Pb, etc) and demonstrate that a ‘complete’ charge transfer (nominally one electron transfer) between the two transition metal ions^13–18^ can induce desirable properties for bulk photovoltaics. First-principles calculations show that while neither bulk perovskite *A*VO_3_ nor *A*FeO_3_ is ferroelectric, a ‘complete’ charge transfer occurs from V to Fe, rendering the new double perovskite oxides a Mott multiferroic: at zero temperature a ferroelectric antiferromagnet and around room temperatures a ferroelectric Mott insulator. The ferroelectric polarization is substantial, comparable to *A*TiO_3_, but the band gap is significantly lower, smaller than that of *A*TiO_3_ and BiFeO_3_.

**Figure 1:**
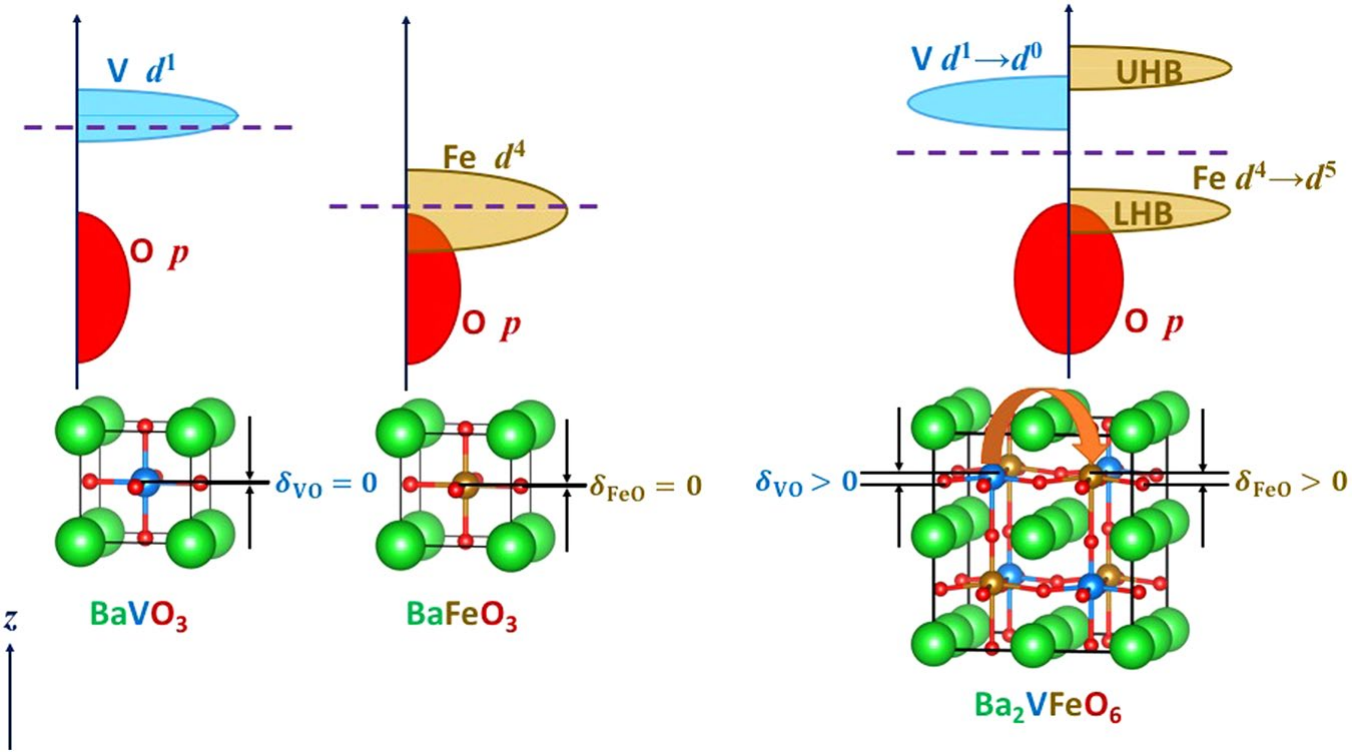
Design principles for charge-transfer-driven Mott multiferroics. (a) Energy diagram and atomic structure of cubic BaVO_3_. The dashed line is the Fermi level. *δ*_VO_ is the V-O displacement along the [001] direction. (b) Energy diagram and atomic structure of cubic BaFeO_3_. The dashed line is the Fermi level. *δ*_FeO_ is the Fe-O displacement along the [001] direction. (c) Energy diagram and atomic structure of double perovskite Ba_2_VFeO_6_. The dashed line is the Fermi level, which lies in the gap between V *d* and Fe *d* states. ‘LHB’ (‘UHB’) means lower Hubbard bands (upper Hubbard bands). The red arrow indicates the charge transfer from V atoms to Fe atoms due to electronegativity difference. In the double perovskite Ba_2_VFeO_6_, a polar distortion is developed (*δ*_VO_ > 0 and *δ*_FeO_ > 0) because of the new charge configuration V *d* ^0^ and Fe *d* ^5^.

We first focus on Ba_2_VFeO_6_ (similar results are obtained for Pb_2_VFeO_6_ and Sr_2_VFeO_6_, see section 4). Figure 1a and b show the atomic and electronic structures for perovskite BaVO_3_ and BaFeO_3_, respectively. Bulk perovskite BaVO_3_ has been recently synthesized at high pressure and has been found to remain cubic and metallic to the lowest temperature^19^. Bulk BaFeO_3_ normally crystallizes in a hexagonal structure but cubic perovskite BaFeO_3_ can be stabilized in powders^20^ and in epitaxial thin films^21–24^ and exhibits a robust ferromagnetism^20–24^. Both metallic^20, 23^ and insulating^21, 22, 24^ behaviors have been reported.

Formal valence considerations imply that in BaVO_3_ the V adopts a *d* ^1^ configuration while in BaFeO_3_ the Fe is *d* ^4^. In the double perovskite Ba_2_VFeO_6_, however, we expect that the large electronegativity difference between V and Fe leads to complete charge transfer from V to Fe, resulting in V-*d* ^0^ and Fe-*d* ^5^ configurations as illustrated in Fig. 1c. Similar phenomena have been predicted and observed in many different transition metal oxide heterostructures^15–18, 25^. The particular relevance here is that the empty V-*d* shell and half-filled Fe-*d* shell are both susceptible to noncentrosymmetric distortions (for the empty *d* shell case, see refs 26 and 27 and for the half-filled *d* shell cases see refs 28–30) while Ba^2+^-O^2−^ coupling stabilizes the ferroelectric phase over anti-ferroelectric phases, as in BaTiO_3_^31^. The half filled Fe-*d* shell leads to magnetic ordering and Mott insulating behavior, while the position of the V-*d* level leads to a reduced band gap (a similar strategy to reduce band gap has been discussed in refs 12, 26, 27. Therefore as Fig. 1c shows, double perovskite Ba_2_VFeO_6_ is predicted to be Mott multiferroic (paramagnetic ferroelectric at high temperatures and long-range magnetically ordered at sufficiently low temperatures). Furthermore, as illustrated in Fig. 1c, the band gap of double perovskite Ba_2_VFeO_6_ is set by the filled lower Hubbard band of Fe-*d* states (strongly hybridized with O-*p* states) and empty V-*d* states (conduction band edge).

We note that the double perovskite structure is much more stable than the layered configuration proposed in ref. 12, because charge transfer generically results in substantial metal-oxygen bond disproportionation^25^. Due to geometry consideration, the bond disproportionation inevitably induces internal strain in the layered structure but is naturally accommodated by the double perovskite structure, explaining the relative phase stability^25^. Although previous work has suggested that rock-salt ordering of *B*-site atoms suppresses polarization in *A*_2_ *BB*′O_6_^12, 32^, our work shows that it is possible to induce robust ferroelectricity in double perovskite oxides Ba_2_VFeO_6_.

In the rest of this paper we present calculations substantiating this picture. In Section II we outline the computational details. In Section III we present results for double perovskite Ba_2_VFeO_6_. Section IV extends the calculations to the double perovskite Pb_2_VFeO_6_ and Sr_2_VFeO_6_, in which we discuss the similarities and differences. Section V is a summary and conclusion.

## Computational Details

Our first-principles calculations are performed using density functional theory (DFT)^33^ and dynamical mean field theory (DMFT)^34^. Structural relaxation is performed within DFT. Gaps are calculated using both DFT and DFT+DMFT. It has been established that structural and magnetic properties of multiferroic oxides strongly depend on the choice of exchange correlation functionals^5, 30, 35^. We use three exchange correlation functionals to test the robustness of our predictions: i) charge-density-only generalized gradient approximation with Perdew-Burke-Ernzerhof parametrization^36^ plus Hubbard *U* and Hund’s *J* corrections (PBE+*U*+*J*)^37^, (ii) charge-only local density approximation with Hubbard *U* and Hund’s *J* corrections (LDA+*U*+*J*)^37, 38^; (iii) spin-polarized generalized gradient approximation with Perdew-Burke-Ernzerhof parametrization revised for solids (sPBEsol)^39^. In order to investigate Mottness and effects of long-range magnetic ordering, we use DMFT to study both paramagnetic and long-range magnetic ordered states.

The DFT calculations are performed using a plane-wave basis^33^, as implemented in the Vienna Ab-initio Simulation Package (VASP)^40, 41^. The Projector Augmented Wave (PAW) approach is used^42, 43^. We use an energy cutoff of 600 eV. All the supercells of double perovskite oxides *A*_2_VFeO_6_ consist of 40 atoms to accommodate different magnetic orderings. We consider ferromagnetic ordering, [001] antiferromagnetic ordering, [010] antiferromagnetic ordering and [100] antiferromagnetic ordering (see the Supplementary Materials for their definitions). We note that since in *A*_2_VFeO_6_ the Fe ions form a face-centered-cubic lattice which has intrinsic ‘geometry frustration’, novel magnetism such as non-collinear magnetic ordering is possible in the ground state^44, 45^. However, at finite temperatures, [001] antiferromagnetic ordering has been observed in various double perovskite oxides^46–49^. In this study, we only consider collinear magnetic orderings. A 6 × 6 × 6 Monkhorst-Pack grid is used to sample the Brillouin zone. Both cell and internal coordinates are fully relaxed until each force component is smaller than 10 meV/Å and the stress tensor is smaller than 0.1 kbar.

In the PBE+*U*+*J*/LDA+*U*+*J* as well as DMFT calculations, we use *U*_Fe_ = 5 eV, *J*_V_ = *J*_Fe_ = 0.7 eV, following previous studies^50, 51^. The choice of *U*_V_ needs comment. While *U*_V_ of about 5 eV has been accepted in literature^50^, we find that *U*_V_ = 5 eV induces an off-center displacement *δ*_VO_ in perovskite BaVO_3_, while in experiment perovskite BaVO_3_ (which experimentally is stable only at pressures P > 15GPa) is a cubic structure^19^. The calculated off-center displacement of V is closely related to orbital ordering (${d}_{xy}^{1}{d}_{xz}^{0}{d}_{yz}^{0}$) stabilized by a large *U*_V_ in the DFT+*U* method. Therefore we use a smaller *U*_V_ = 3 eV which stabilizes a cubic structure in perovskite BaVO_3_  in our calculations of double perovskite Ba_2_VFeO_6_. This ensures that a non-zero *δ*_VO_ in Ba_2_VFeO_6_ is not a consequence of a large *U*_V_, but rather is induced by charge transfer. We repeated all the DFT calculations on Ba_2_VFeO_6_ using *U*_V_ = 5 eV and found qualitatively similar results in structural properties. On the other hand, *U*_V_ controls the energy level of V-*d* states, which may affect the band gap of Ba_2_VFeO_6_. Therefore, in our DMFT calculations, we also studied a range of *U*_V_ (from 3 to 6 eV) to estimate the variation of energy gap in the spectral function.

We perform single-site DMFT calculations with Ising-like Slater-Kanamori interactions. The impurity problem is solved using the continuous-time quantum Monte Carlo algorithm with a hybridization expansion^52, 53^. The correlated subspace and the orbitals with which it mixes are constructed using maximally localized Wannier functions^54^ defined over the full 10 eV range spanned by the *p*-*d* band complex, resulting in a well-localized set of *d*-like orbitals. All the DMFT calculations are performed at the temperature of 290 K. For each DMFT iteration, a total of 3.8 billion Monte Carlo steps is taken to converge the impurity Green function and self energy. In double perovskite oxides, since V-*d* states are empty, we treat V-*t*_2*g*_ orbitals with the DMFT method and V-*e*_*g*_ orbitals with a static Hartree-Fock approximation. Because the Fe-*d* states are half-filled, we treat all the five Fe-*d* orbitals with the DMFT method. The two self energies (one for V sites and the other for Fe sites) are solved independently and then coupled at the level of self-consistent conditions.

To obtain the spectral functions, the imaginary axis self energy is continued to the real axis using the maximum entropy method^55^. Then the real axis local Green function is calculated using the Dyson equation and the spectral function is obtained following:

1 $${A}_{i}(\omega )=-\frac{1}{\pi }{\rm{Im}}{G}_{i}^{{\rm{loc}}}(\omega )=-\frac{1}{\pi }{\rm{Im}}{(\sum _{{\rm{k}}}\frac{1}{(\omega +\mu ){\bf{1}}-{H}_{0}({\bf{k}})-{\rm{\Sigma }}(\omega )})}_{ii}$$

where *i* is the label of a Wannier function. **1** is an identity matrix, *H*_0_(**k**) is the DFT-PBE band Hamiltonian in the matrix form using the Wannier basis. Σ(*ω*) is understood as a diagonal matrix only with nonzero entries on the correlated orbitals. *μ* is the chemical potential. *V*_*dc*_ is the fully localized limit (FLL) double counting potential, which is defined as in ref. 56:

2 $${V}_{dc}=(U-2J)({N}_{d}-\frac{1}{2})-\frac{1}{2}J({N}_{d}-3)$$

where *N*_*d*_ is the *d* occupancy of a correlated site.

## Results for Ba_2_VFeO_6_

### Structural properties

We first discuss the fully relaxed atomic structure of double perovskite Ba_2_VFeO_6_, obtained using DFT calculations with three different exchange correlation functionals (PBE+*U*+*J*, LDA+*U*+*J* and sPBEsol). For each exchange correlation functional, we test ferromagnetic (*F*), [001] antiferromagnetic, [010] antiferromagnetic and [100] antiferromagnetic orderings (see the Supplementary Materials for precise definitions). For each case, we start from a crystal structure with rotations and tilts of VO_6_ and FeO_6_ (space group *P*2_1_/*n*) and then perturb the V and Fe atoms along [001] or [011] or [111] directions. Next we perform atomic relaxation with all the symmetry turned off. After atomic relaxation, we find that the rotations and tilts of VO_6_ and FeO_6_ are strongly suppressed while the polarization along [001] or [011] or [111] direction is stabilized. Comparing the total energy of the three polarizations, we find the ground state of Ba_2_VFeO_6_ has the polarization along the [001] direction. The ground state structure has tetragonal symmetry (space group *I*4/*m*). We note that based on the symmetry analysis^57^ and all the available experimental data for double perovsite oxides compiled in the review^49^, there are altogether seven tilting patterns which are allowed in a double perovskite structure *A*_2_ *BB*′O_6_ and have been observed in experiment. They are: *a* ^0^ *a* ^0^ *a* ^0^(*Fm*-3*m*), *a* ^+^ *b* ^−^ *b* ^−^(*P*2_1_/*n*), *a* ^0^ *a* ^0^ *c* ^−^(*I*4/*m*), *a* ^−^ *a* ^−^ *a* ^−^(*R*-3), *a* ^0^ *b* ^−^ *b* ^−^(*I*2/*m*), *a* ^0^ *a* ^0^ *c* ^+^(*P*4/*mnc*) and *a* ^−^ *b* ^−^ *c* ^−^(*I*-1) (the last two tilting patterns are much rarer in experiment). Among them, the most common tilting is *a* ^+^ *b* ^−^ *b* ^−^(*P*2_1_/*n*) with over 300 compounds^49^. We tested different initial guesses with these and other allowed symmetries, perturbed the system with ferroelectric distortions, and after relaxation we always obtained similar results. On the magnetic properties, given the *U* and *J* values, we find that the ground state is always of the [001] antiferromagnetic ordering (among the collinear magnetic orderings). Using the same methods and parameters, perovskite BaVO_3_ and BaFeO_3_ have cubic symmetry. The resulting lattice constant *a*, tetragonality *c*/*a* ratio and cation-displacement *δ*_*BO*_ along the [001] direction (see in Fig. 1c) are shown in Table 1 for each exchange correlation functional. The full crystal structure data are provided in the Supplementary Materials. We observe that the reason that rotations and tilts of VO_6_/FeO_6_ octahedra are strongly suppressed in Ba_2_VFeO_6_ is due to the large ionic size of Ba ions, which is known to prohibit rotations and tilts of oxygen octahedra in perovskite Ba-compounds and to induce robust ferroelectricity in BaTiO_3_ and BaMnO_3_^29, 58^.

**Table 1:** Comparison of Ba_2_VFeO_6_ and BaTiO_3_.

	Ba_2_VFeO_6_			BaTiO_3_		
xc	PBE+*U*+*J*	LDA+*U*+*J*	sPBEsol	PBE	LDA	sPBEsol
magnetic	[001]	[001]	[001]	nm	nm	nm
	cubic structure					
*a* (Å)	4.016	3.922	3.965	4.036	3.952	3.991
Δ_0_ (eV)	0.55	0.35	0.45	1.70	1.70	1.80
	tetragonal structure					
*a* (Å)	3.958	3.916	3.946	4.001	3.944	3.978
*c*/*a*	1.078	1.007	1.024	1.053	1.011	1.021
*δ*_*B*O_ (Å)	0.195 (V)	0.067 (V)	0.116 (V)	0.197	0.099	0.133
	0.265 (Fe)	0.086 (Fe)	0.152 (Fe)			
*P* (*μ*C/cm^2^)	50	18	34	46	23	33
Δ_0_ (eV)	0.78	0.38	0.59	1.75	1.75	1.75
Δ_optical_ (eV)	1.10	1.04	1.17	2.30	2.02	2.14
Δ*E* (meV)	−43	−1	−7	−56	−6	−17
*m* (*μ*_*B*_)	0.129 (V)	0.071 (V)	0.091 (V)	—	—	—
	4.023 (Fe)	4.075 (Fe)	4.063 (Fe)			

The results are calculated using the DFT method with different exchange correlation functionals (xc). ‘nm’ stands for non-magnetic and ‘[001]’ for [001] antiferromagnetic ordering. For the cubic case, *a* is the lattice constant and Δ_0_ is the fundamental gap. For the tetragonal case, *a* is the in-plane lattice constant, *c*/*a* is the ratio of out-of-plane lattice constant over in-plane lattice constant, *δ*_*B*O_ is the *B*-site metal and oxygen displacement along the [001] direction. Δ_0_ is the fundamental gap and Δ_optical_ is the optical gap. Δ*E* is the energy difference between the tetragonal structure and the cubic structure in the unit of meV per 5-atom formula. *P* is the polarization along the [001] direction. *m* is the local magnetic moment on V and Fe sites.

For comparison, we also calculated the atomic structure of fully relaxed tetragonal BaTiO_3_, a known ferroelectric perovskite. Since BaTiO_3_ is a *d* ^0^ band insulator with no magnetic properties, we do not add Hubbard *U* and Hund’s *J* correction to PBE/LDA and we use PBEsol instead of spin-polarized PBEsol (sPBEsol). We find that the calculated *c*/*a* ratio and ion-displacement (*δ*_VO_ and *δ*_FeO_) of Ba_2_VFeO_6_ are comparable to those of BaTiO_3_. The ground state of tetragonal double perovskite Ba_2_VFeO_6_ is an insulator (we will discuss the gap properties in details in the following subsections). The ground state of high-symmetry cubic double perovskite Ba_2_VFeO_6_ is also an insulator (see Table 1). Therefore a switching path for ferroelectric polarization is well-defined and we can use the Berry phase method^54^ to calculate the polarization of the tetragonal structure. We find that for each exchange-correlation function the calculated polarization of Ba_2_VFeO_6_ is comparable to that of BaTiO_3_ (see Table 1).

We comment here that our recent studies^30, 35^ of perovskite manganites show that PBE+*U*+*J* and sPBEsol yield the most accurate predictions of structural and magnetic properties of magnetic ferroelectrics, while LDA+*U*+*J* sets a conservative estimation for the lower bound of polarization. Therefore we believe that the polarization of Ba_2_VFeO_6_ is larger than 18 *μ*C/cm^2^, which is substantial enough to induce bulk photovoltaic effects^4^.

### Electronic properties

The results of the previous subsection indicate that the double perovskite Ba_2_VFeO_6_ has a noncentrosymmetric tetragonal distortion not found in the component materials bulk BaVO_3_ and BaFeO_3_. In this section we consider the electronic reconstruction arising in this double perovskite.

**Figure 2:**
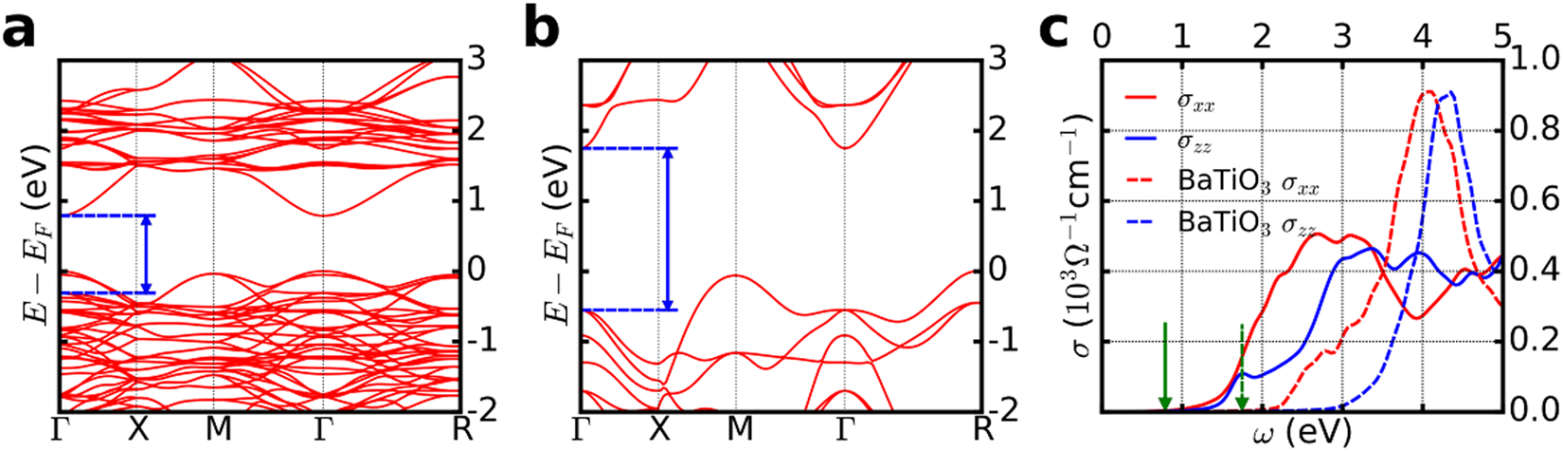
Comparison of band structure and optical conductivity between Ba_2_VFeO_6_ and BaTiO_3_. The results for Ba_2_VFeO_6_ are calculated using DFT-PBE+*U*+*J* method. The results for BaTiO_3_ are calculated using DFT-PBE method. (**a**) Band structure of tetragonal Ba_2_VFeO_6_. The blue arrow indicates the threshold of optical transition. (**b**) Band structure of tetragonal BaTiO_3_. The blue arrow indicates the threshold of optical transition. (**c**) Optical conductivity *σ* of tetragonal Ba_2_VFeO_6_ (solid lines) and tetragonal BaTiO_3_ (dashed lines). The red lines are for the *xx*-component and the blue lines are for the *zz*-component. The green arrows indicate the fundamental gap of band structures.

Figure 2a shows the band structure of double perovskite Ba_2_VFeO_6_ with the [001] antiferromagnetic ordering (only one spin channel is shown here), calculated using the PBE+*U*+*J* method. We see that a gap is clearly opened in Ba_2_VFeO_6_ while using the same method with the same parameters, perovskite BaVO_3_ and BaFeO_3_ are found to be metallic with V-*d* and Fe-*d* states at the Fermi surface (see Section II in the Supplementary Materials for details). The gap opening in Ba_2_VFeO_6_ is a strong evidence of a nominally “complete” charge transfer from V to Fe. A similar charge-transfer-driven metal-insulator transition is predicted^14^ and observed^17^ in LaTiO_3_/LaNiO_3_ superlattices.

For comparison, we also calculated the band structure of tetragonal BaTiO_3_ using PBE (Fig. 2b). We note that while the polarization of double perovskite Ba_2_VFeO_6_ is comparable to that of BaTiO_3_, the band gap of Ba_2_VFeO_6_ (0.78 eV) is significantly smaller than that of BaTiO_3_ (1.75 eV). Using other exchange correlation functionals, we find similar properties that the band gap of Ba_2_VFeO_6_ is smaller than that of BaTiO_3_ by about 1 eV (see ‘fundamental gap’ Δ_0_ in Table 1).

For photovoltaic effects the relevant quantity is the optical gap Δ_optical_. We calculate the optical conductivity of both Ba_2_VFeO_6_ and BaTiO_3_ using standard methods^59^ and show the results in Fig. 2c. Due to the tetragonal symmetry, the off-diagonal matrix elements of the optical conductivity vanish and only two diagonal elements are independent (*σ*_*xx*_ = *σ*_*yy*_ and *σ*_*zz*_). For BaTiO_3_ the minimum optical gap is in the *xx* channel and is given by the direct (vertical in momentum space) gap (shown for BaTiO_3_ as the blue arrow in Fig. 2b). In BaTiO_3_ the optical gap is larger than the fundamental gap, which is indirect (momentum of lowest conduction band state differs from momentum of highest valence band state; the green arrow in Fig. 2c shows the size of the fundamental gap). The optical conductivity of Ba_2_VFeO_6_ is also larger than its fundamental gap, which can be understood in a similar manner. If we consider (VFe) as a pseudo-atom *X*, the hypothetical single perovskite Ba*X*O_3_ would have an indirect gap (between Γ and *R*). However, the reduction in translational symmetry due to the V-Fe alternation leads to band folding which maps the original *R* point to the Γ point, leading to a direct gap of 0.8 eV at the Γ point. However the calculated optical gap is 1.1 eV (blue arrow in Fig. 2a). The difference between the direct and optical gaps is a matrix element effect: the lowest back-folded conduction band state does not have a dipole allowed transition matrix element with the highest-lying valence band state (see the Supplementary Materials for more details).

It is well-known that DFT with semi-local exchange correlation functionals substantially underestimate band gaps. Here we argue that since Ba_2_VFeO_6_ and BaTiO_3_ have similar electronic structures (gap separated by metal *d* and oxygen *p* states), the DFT band gap underestimation with respect to experimental values is approximately a constant for BaTiO_3_ and Ba_2_VFeO_6_. The experimental optical gap of BaTiO_3_ is 3.2 eV and the DFT calculated value is 2.3 eV, about 0.9 eV smaller. The DFT calculated optical gap of Ba_2_VFeO_6_ is 1.1 eV, hence we estimate the experimental optical gap of Ba_2_VFeO_6_ is 2.0 eV, which is smaller than the optical gap of intensively investigated BiFeO_3_ (2.7 eV)^60^.

**Figure 3:**
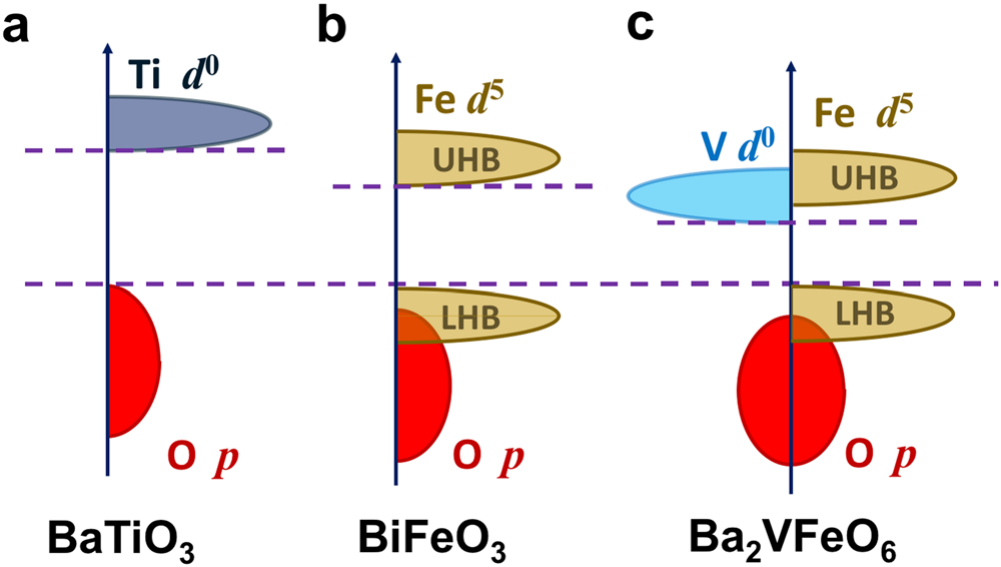
Comparison of gaps for different perovskite oxides. (**a**) BaTiO_3_; (**b**) BiFeO_3_; (**c**) Ba_2_VFeO_6_. ‘LHB’ (‘UHB’) means lower Hubbard bands (upper Hubbard bands). The valence band edges are aligned for comparison.

Our results that Ba_2_VFeO_6_ should have a smaller gap than that of BaTiO_3_ and BiFeO_3_ are also supported by physical arguments (see Fig. 3). The band gap for transition metal oxides is set by the energy difference between transition metal *d* states and oxygen *p* states. This *p*-*d* separation is a measure of the relative electronegativity of transition metal and oxygen ions. Ti and V are both first-row transition metals and in BaTiO_3_ and Ba_2_VFeO_6_, Ti and V both have a *d* ^0^ configuration. Because V has a larger nuclear charge than Ti, the V-*d* states have lower energies than the Ti-*d* states, which leads to a smaller band gap for Ba_2_VFeO_6_ than for BaTiO_3_ (compare panels a and c of Fig. 3). On the other hand, the Fe *d* states are half-filled in both Ba_2_VFeO_6_ and BiFeO_3_, while V-*d* states are empty in Ba_2_VFeO_6_. Due to Coulomb repulsion and Hund’s coupling effects, adding one more electron in a half-filled *d* shell generically costs more energy than adding an electron in an empty *d* shell. Therefore the upper Hubbard band of Fe *d* states have higher energy than V *d* states, which results in a larger band gap for BiFeO_3_ than for Ba_2_VFeO_6_ (compare panels b and c of Fig. 3).

### Estimation of critical temperatures

Double perovskite Ba_2_VFeO_6_ is a type-I multiferroic^61^, in which ferroelectric polarization and magnetism arise from different origins and they are largely independent of one another. This means that ferroelectric polarization and magnetism have their own critical temperatures and usually the critical temperature of polarization (*T*_*C*_) is higher than the critical temperature of magnetism (*T*_*N*_)^62^. In this subsection, we estimate *T*_*C*_ and *T*_*N*_ for Ba_2_VFeO_6_.

#### Estimation of *T*_*C*_

In order to estimate the ferroelectric Curie temperature *T*_*C*_, we use the predictor ${T}_{C}\propto {P}_{0}^{2}$ where *P*_0_ is the zero-temperature polarization^63^. This predictor has been successfully applied to a wide range of Pb-based perovskite ferroelectric oxides and it yields an accurate and quantitative estimation for ferroelectric *T*_*C*_^64^. We apply this predictor to our Ba-based ferroelectrics, i.e. BaTiO_3_ and Ba_2_VFeO_6_. Here we use tetragonal BaTiO_3_ as the reference system. The experimental Curie temperature *T*_*C*_ for BaTiO_3_ is about 400 K^65^. Using the DFT+Berry phase method^54^, we can obtain the values of the zero-temperature polarization for both BaTiO_3_ and Ba_2_ VFe _6_ shown in Table 1. Therefore we estimate that *T*_*C*_ for Ba_2_VFeO_6_ is 473 K (PBE+*U*+*J*), 245 K (LDA+*U*+*J*) and 425 K (sPBEsol). While different exchange correlation functionals predict a range for *T*_*C*_, we find that *T*_*C*_ is near or above room temperature.

#### Estimation of *T*_*N*_

We use a classical Heisenberg model $E=\frac{1}{2}{\sum }_{\langle kl\rangle }{J}_{kl}{{\bf{S}}}_{k}\cdot {{\bf{S}}}_{l}$ to estimate the magnetic ordering transition temperature *T*_*N*_, where **S**_*k*_ is a unit-length classical spin and 〈*kl*〉 denotes summation over nearest Fe neighbors. Here we only consider Fe-Fe exchange couplings. Because double perovskite Ba_2_VFeO_6_ has a tetragonal structure, there are two exchange couplings of *J*_*kl*_: *J*_in_ for the short Fe-Fe bonds and *J*_out_ for the long Fe-Fe bonds. By calculating the total energy for the ferromagnetic ordering, [001] antiferromagnetic ordering and [100] antiferromagnetic ordering, we obtain that the in-plane exchange coupling *J*_in_ is 2.5 meV (PBE+*U*+*J*), 3.7 meV (LDA+*U*+*J*) and 3.1 meV (sPBEsol); and the out-of-plane exchange coupling *J*_out_ is 3.1 meV (PBE+*U*+*J*), 4.0 meV (LDA+*U*+*J*) and 3.7 meV (sPBEsol). The positive sign means that exchange couplings are all antiferromagnetic. Based on a mean-field theory, the estimated Néel temperature is *T*_*N*_ = |4*J*_in_ − 8*J*_out_|. The minus sign is because on a quasi face-centered-cubic lattice, every Fe atom has 8 nearest neighbors that are antiferromagnetically coupled and 4 nearest neighbors that are ferromagnetically coupled. Therefore *T*_*N*_ is estimated to be 172 K (PBE+*U*+*J*), 200 K (LDA+*U*+*J*) and 200 K (sPBEsol). Since mean-field theories usually overestimate magnetic transition temperatures, the actual *T*_*N*_ could be lower. An experimental determination of the magnetic ordering temperature would be of great interest.

### Effects of long-range order

The estimates for the ferroelectric and magnetic transition temperatures of Ba_2_VFeO_6_ suggest that its actual ferroelectric Curie temperature *T*_*C*_ is probably higher than its actual Néel temperature *T*_*N*_, as is the case for most type-I multiferroics^61^. It is therefore important to ask if the magnetically disordered state remains insulating, so that the ferroelectric properties are preserved.

**Figure 4:**
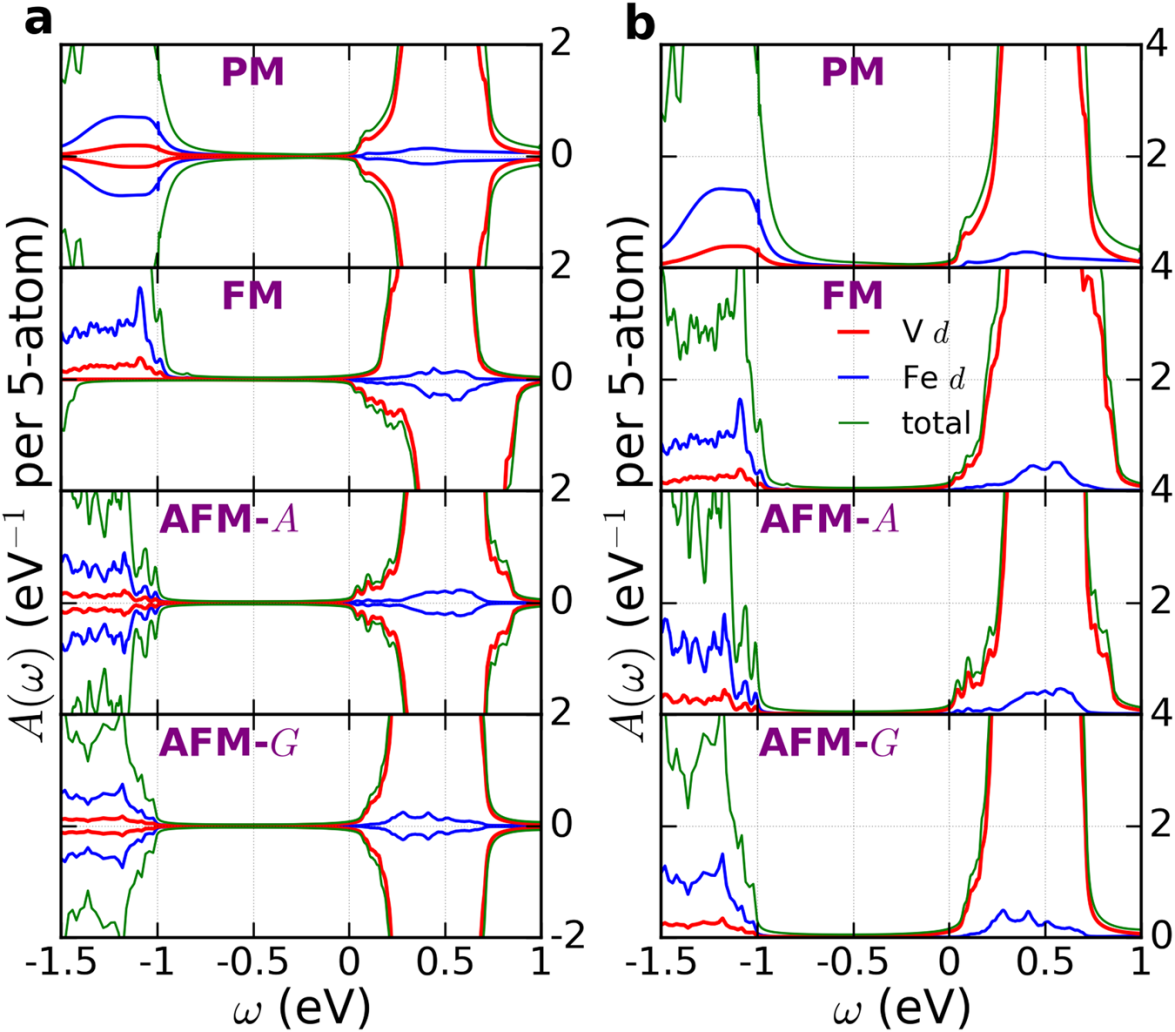
Spectral functions *A*(*ω*) of tetragonal double perovskite Ba_2_VFeO_6_ for different magnetic states. The unit of *A*(*ω*) is eV^−1^ per 5-atom. ‘PM’ stands for paramagnetic state, ‘FM’ for ferromagnetic state, ‘[001]-AFM’ for [001] antiferromagnetic state and ‘[100]-AFM’ for [100] antiferromagnetic state. Panels a) spin-resolved spectral function. The positive (negative) *y*-axis corresponds to spin-up (spin-down). Panels b) total spectral functions (summing over spin-up and spin-down). The red, blue and green curves are for Fe *d*, V *d* and O *p*, respectively. The Fermi level is set at *ω* = 0 eV.

Here we use DFT+DMFT to study both the paramagnetic and magnetically ordered states. The spectral functions for the three magnetic states that we have considered are shown in Fig. 4 along with the spectral function for the paramagnetic state. We find that the paramagnetic state is insulating, with a gap only slightly smaller than that of the ground state with [001] antiferromagnetic ordering, indicating that double perovskite Ba_2_VFeO_6_ is a promising candidate for Mott multiferroics^62^. The calculated spectral functions are consistent with our schematics of Fig. 3.

**Figure 5:**
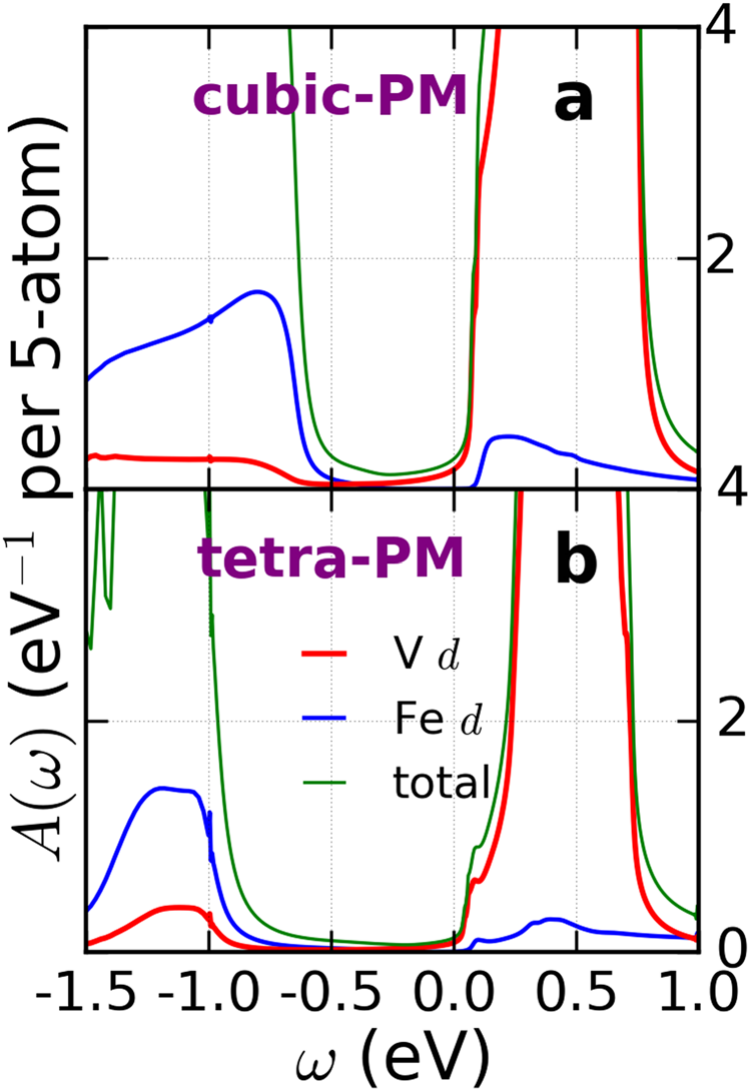
Spectral functions *A*(*ω*) of cubic and tetragonal Ba_2_VFeO_6_. The unit of *A*(*ω*) is eV^−1^ per 5-atom. Panel a is for cubic Ba_2_VFeO_6_ and panel b is for tetragonal Ba_2_VFeO_6_. In both structures, we calculate the paramagnetic state. The red, blue and green curves are for Fe *d*, V *d* and O *p*, respectively. The Fermi level is set at *ω* = 0 eV.

We also use our DFT+DMFT methodology to investigate how the electronic structure of Ba_2_VFeO_6_ evolves as the ferroelectric polarization is suppressed within the paramagnetic state. Figure 5 compares the spectral function of Ba_2_VFeO_6_ in the cubic structure (i.e. no polarization) versus in the tetragonal structure (i.e. with polarization). We see that the suppression of polarization reduces the gap by about 0.2 eV. This behavior is very consistent with similar calculations on the nonmagnetic perovskite oxide SrTiO_3_ in which the presence of ferroelectric polarization can increase the band gap by up to 0.2 eV^66^.

### Hubbard *U* dependence

**Figure 6:**
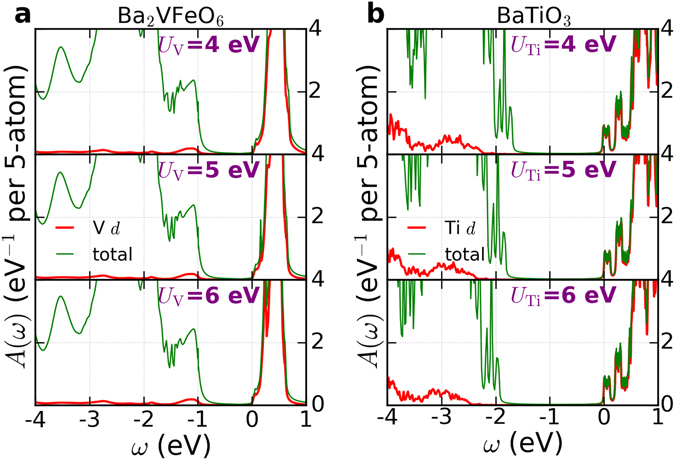
Spectral functions *A*(*ω*) of tetragonal Ba_2_VFeO_6_ and BaTiO_3_ as a function of Hubbard *U* on V and Ti. The unit of *A*(*ω*) is eV^−1^ per 5-atom. Panels a are the results for tetragonal Ba_2_VFeO_6_. Panels b are the results for tetragonal BaTiO_3_. For Ba_2_VFeO_6_, the calculations are performed in a paramagnetic state. For BaTiO_3_, the calculations are performed in a non-magnetic state. In panels a, the green lines are the total spectral functions and the red lines are the spectral functions projected onto V *d* states. In panels b, the green lines are the total spectral functions and the red lines are the spectral functions projected onto Ti *d* states. The Fermi level is set at *ω* = 0 eV.

Finally we discuss the Hubbard *U* dependence. As Fig. 4 shows, the conduction band edge is set by V-*d* states, which is consistent with Fig. 1c and our previous discussion of band gaps. If we change the Hubbard *U*_V_, it may affect the energy position of V *d* states and energy gap. To address this issue, we repeat the DMFT calculations on tetragonal Ba_2_VFeO_6_ using several values of *U*_V_. The panels a of Fig. 6 show the spectral function of the double perovskite as a function of *U*_V_. All the calculations are performed in a paramagnetic state. We note that as *U*_V_ increases from 4 eV to 6 eV, the band gap is almost unchanged. This is due to the fully localized limit double counting correction which nearly cancels the Hartree shift. Hence, the V-*d* and O-*p* energy separation is practically unaffected, which is very consistent with a previous DMFT study of SrVO_3_^67^. If we apply the same method and same Hubbard *U* parameters to tetragonal BaTiO_3_, the spectral functions of BaTiO_3_ (panels b of Fig. 6) show that the energy gap of BaTiO_3_ is slightly increased. Thus while we have some uncertainty relating to the appropriate values for the Hubbard *U*, our estimates for energy gap are robust: double perovskite Ba_2_VFeO_6_ has an energy gap ~1 eV smaller than that of BaTiO_3_. The underlying reason is the differing electronegativities of Ti^4+^ and V^5+^.

## Related materials Pb_2_VFeO_6_ and Sr_2_VFeO_6_

In this section we employ the same parameters and methods used for Ba_2_VFeO_6_ to discuss double perovskite Pb_2_VFeO_6_ and Sr_2_VFeO_6_.

We first discuss Pb_2_VFeO_6_. Pb has a lone pair of 6*s* electrons, which favors off-center displacements as was already shown for tetragonal PbTiO_3_^68^. Due to the same mechanism, double perovskite Pb_2_VFeO_6_ has substantial cation-displacements, tetragonality and ferroelectric polarization (see Table 2). All these values are comparable to, or even larger than those of tetragonal PbTiO_3_. We note however that within sPBEsol the polarization of this tetragonal structure is not-well defined because the corresponding high-symmetry cubic structure is metallic and thus the obvious switching path is not available.

**Table 2:** Comparison of Pb_2_VFeO_6_ and PbTiO_3_.

	Pb_2_VFeO_6_			PbTiO_3_		
xc	PBE+*U*+*J*	LDA+*U*+*J*	sPBEsol	PBE	LDA	PBEsol
magnetic	[001]	[001]	[001]	nm	nm	nm
	cubic structure					
*a* (Å)	3.949	3.857	3.887	3.972	3.891	3.929
Δ_0_ (eV)	0.60	0.41	metallic	1.61	1.47	1.53
	tetragonal structure					
*a* (Å)	3.803	3.776	3.751	3.844	3.865	3.882
*c*/*a*	1.248	1.116	1.220	1.238	1.044	1.081
*δ*_*B*O_ (Å)	0.425 (V)	0.281 (V)	0.413 (V)	0.526	0.277	0.346
	0.629 (Fe)	0.463 (Fe)	0.601 (Fe)			
*P* (*μ*C/cm^2^)	124	102	—	125	79	93
Δ_0_ (eV)	0.42	0.38	0.26	1.88	1.49	1.60
Δ_optical_ (eV)	1.83	1.83	1.88	2.86	2.48	2.82
Δ*E* (meV)	−251	−77	−239	−209	−57	−79
*m* (*μ*_*B*_)	0.147 (V)	0.163 (V)	0.183 (V)	—	—	—
	4.004 (Fe)	4.002 (Fe)	3.674 (Fe)			

The results are calculated using the DFT method with different exchange correlation functionals (xc). ‘nm’ stands for non-magnetic and ‘[001]’ for the [001] antiferromagnetic ordering. For the cubic case, *a* is the lattice constant and Δ_0_ is the fundamental gap. For the tetragonal case, *a* is the in-plane lattice constant, *c*/*a* is the ratio of out-of-plane lattice constant over in-plane lattice constant, *δ*_*B*O_ is the *B*-site metal and oxygen displacement along the [001] direction. Δ_0_ is the fundamental gap and Δ_optical_ is the optical gap. Δ*E* is the energy difference between the tetragonal structure and the cubic structure in the unit of meV per 5-atom formula. *P* is the polarization along the [001] direction. *m* is the local magnetic moment on V and Fe sites.

While tetragonal double perovskite Pb_2_VFeO_6_ has similar structural properties to tetragonal PbTiO_3_, the fundamental gap Δ_0_ and optical gap Δ_optical_ are both smaller than those of PbTiO_3_ by about 1 eV (all three exchange correlation functionals make qualitatively consistent predictions).

We note here that the polarization in Pb_2_VFeO_6_ has different origin from the polarization in tetragonal PbVO_3_^69^. In tetragonal PbVO_3_, V atoms have a *d* ^1^ charge configuration and its off-center displacement *δ*_VO_ and insulating properties are associated with orbital ordering (${d}_{xy}^{1}{d}_{xz}^{0}{d}_{yz}^{0}$)^70^. In double perovskite oxide Pb_2_VFeO_6_, charge transfer leads to a *d* ^0^ configuration on V sites and therefore the off-center displacement *δ*_VO_ is due to hybridization between V-*d* and O-*p* states^31^. More importantly, perovskite PbVO_3_ is not ferroelectric because along the switching path (from the tetragonal-to-cubic structure) an insulator-to-metal phase transition is observed^71^.

Next we discuss Sr_2_VFeO_6_. Sr_2_VFeO_6_ is more complicated because the ionic size of Sr^2+^ is smaller than Ba^2+^ and therefore rotations of oxygen octahedra (so-called antiferrodistortive mode, or AFD mode) can exist in Sr-compounds, such as in SrTiO_3_. These rotations compete against ferroelectric polarization^72^. For double perovskite Sr_2_VFeO_6_, even if we do not take the AFD mode into account, different exchange correlation functionals predict different structural and electronic properties. Table 3 shows that PBE+*U*+*J* predicts that the ground state is tetragonal and ferroelectric. The polarization is sizable (26 *μ*C/cm^2^) and the DFT-calculated optical gap is 1.36 eV. On the other hand, the LDA+*U*+*J* method can not stabilize the tetragonal structure. This method predicts that ground state of Sr_2_VFeO_6_ has a cubic structure with no off-center displacements of either V or Fe, and is metallic. The sPBEsol method can stabilize a tetragonal structure with non-zero off-center displacements *δ*_VO_ and *δ*_FeO_, but the ground state is also metallic and therefore the polarization is not well-defined. We may impose epitaxial strain to induce ferroelectricity in Sr_2_VFeO_6_, but the critical strain strongly depends on the choice of exchange correlation functional^30^: PBE+*U*+*J* does not require any strain to stabilize the ferroelectric state, while LDA+*U*+*J* requires a 3% compressive strain to open the gap and stabilize the tetragonal structure with a sizable polarization. A similar situation occurs for SrTiO_3_. If we use the same methods and do not take into account the AFD mode, PBE predicts a ferroelectric ground state, while LDA and sPBE predict that the ground state is cubic (i.e. no polarization). Experimentally, SrTiO_3_ is on the verge of a paraelectric-to-ferroelectric transition^73^. Thus we conclude that our DFT calculations indicate that double perovskite Sr_2_VFeO_6_ is close to the paraelectric-to-ferroelectric phase boundary and probably is on the paraelectric side.

**Table 3:** Comparison of Sr_2_VFeO_6_ and SrTiO_3_.

	Sr_2_VFeO_6_			SrTiO_3_		
xc	PBE+*U*+*J*	LDA+*U*+*J*	sPBEsol	PBE	LDA	PBEsol
magnetic	[001]	[001]	[001]	nm	nm	nm
	cubic structure					
*a* (Å)	3.915	3.823	3.853	3.944	3.863	3.903
Δ_0_ (eV)	0.40	metallic	metallic	1.79	1.80	1.81
	tetragonal structure					
*a* (Å)	3.904	—	3.841	3.936	—	—
*c*/*a*	1.013	—	1.017	1.011	—	—
*δ*_*B*O_ (Å)	0.109 (V)	—	0.181 (V)	0.120	—	—
	0.120 (Fe)		0.162 (Fe)			
*P* (*μ*C/cm^2^)	26	—	metallic	30	—	—
Δ_0_ (eV)	0.30	—	metallic	1.82	—	—
Δ_optical_ (eV)	1.36	—	metallic	2.34	—	—
Δ*E* (meV)	−2	0	−34	−6	0	0
*m* (*μ*_*B*_)	0.084 (V)	0.061 (V)	0.113 (V)	—	—	—
	4.089 (Fe)	4.107 (Fe)	3.543 (Fe)			

The results are calculated using the DFT method with different exchange correlation functionals (xc). Antiferrodistortive modes are not taken into account in the calculations. ‘nm’ stands for non-magnetic and ‘[001]’ for the [001] antiferromagnetic ordering. For the cubic case, *a* is the lattice constant and Δ_0_ is the fundamental gap. For the tetragonal case, *a* is the in-plane lattice constant, *c*/*a* is the ratio of out-of-plane lattice constant over in-plane lattice constant, *δ*_*B*O_ is the *B*-site metal and oxygen displacement along the [001] direction. Δ_0_ is the fundamental gap and Δ_optical_ is the optical gap. Δ*E* is the energy difference between the tetragonal structure and the cubic structure in the unit of meV per 5-atom formula. *P* is the polarization along the [001] direction. *m* is the local magnetic moment on V and Fe sites.

## Conclusions

In summary, we use first-principles calculations to design a new class of Mott multiferroics among which double perovskite oxide Ba_2_VFeO_6_ stands out as a promising candidate to induce bulk photovoltaic effects because of its large polarization (comparable to BaTiO_3_); its reduced optical gap (smaller than BaTiO_3_ by about 1 eV); and its environmentally friendly composition (Pb-free). Our work shows that charge transfer is a powerful approach to engineering atomic, electronic and magnetic structures in complex oxides. New charge configurations not found in bulk materials can occur in oxide heterostructures (including complex bulk forms such as double perovskites), and these charge configurations can produce emergent phenomena and properties not exhibited in constituent compounds. In particular, V^5+^ is very rare in single perovskite oxides (probably due to its small ionic size). We hope our theoretical predictions can stimulate further experimental endeavors to synthesize and measure these new multiferroic materials for photovoltaic applications.

## Electronic supplementary material

Supplementary information
