# Supplementary material for: Design of new Mott multiferroics via complete charge transfer: promising candidates for bulk photovoltaics
Source: Sci Rep. 2017 Jul 21;7:6142. doi: 10.1038/s41598-017-06396-5 (PMC5522466; doi:10.1038/s41598-017-06396-5)
Supplement: Supplementary file 1 — Supplementary information [file 41598_2017_6396_MOESM1_ESM.pdf]

Supplementary Materials of "Design of new Mott multiferroics via complete charge transfer: promising candidates for bulk photovoltaics"

Hanghui Chen[1,2] and Andrew Millis[3]

[1]*NYU-ECNU Institute of Physics,*
NYU Shanghai, Shanghai 200062, China

[2]*Department of Physics,*
New York University,
New York, NY 10002, USA

[3]*Department of Physics,*
Columbia University,
New York, NY 10027, USA

(Dated: April 3, 2017)

I. LONG-RANGE MAGNETIC ORDERING FOR DOUBLE PEROSKITE A_2VFeO$_6$

In double perovskite A_2VFeO$_6$ (A=Ba, Pb, etc), V has a nominally d^0 state and therefore there are no local magnetic moments on V sites. Fe has a nominally d^5 state, which leads a $S = \frac{5}{2}$ local magnetic moment on Fe sites. In double perovskite, we study four long-range magnetic orderings, which are schematically shown in Fig. 1. Note in the cubic structure (space group Fm-$3m$), [001], [010] and [100] antiferromagnetic orderings are all equivalent. In the tetragonal structure (space group $I4/m$), [010] and [100] antiferromagnetic orderings are equivalent.

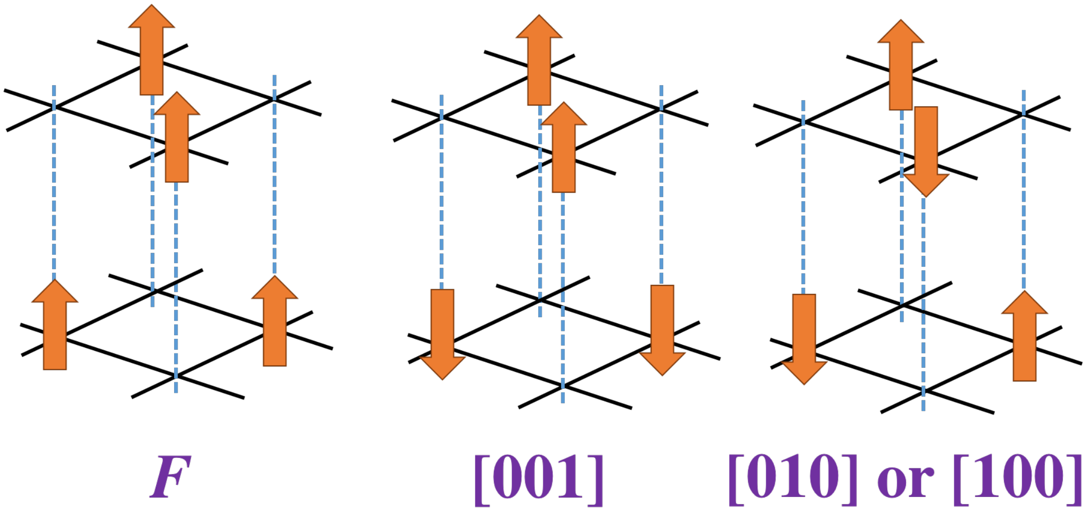

FIG. 1: Different long-range magnetic ordering in double perovskite A_2VFeO$_6$.

II. BULK PROPERTIES OF PEROVSKITE $BaVO_3$ AND $BaFeO_3$

Bulk perovskite $BaVO_3$ is a cubic metal. We consider four common magnetic orderings: ferromagnetic (F), two-sublattice Néel ordering (G), (1, 0, 0) stripe ordering (A) and (1, 1, 0) stripe ordering (C). For each magnetic ordering, we consider a cubic structure in which there is no off-center displacement δ_{VO} in the VO_2 layer (see Fig. 1**a** in the main text), and a tetragonal structure in which there is a nonzero δ_{VO}. We calculate the energy difference ΔE between the two structures. Panels **a1** and **a2** of Fig. 2 show that as $U_{\text{V}} = 3$ eV, for both PBE+U+J and LDA+U+J methods and all the magnetic orderings considered, the ground state is cubic. However, as we increase U_{V}, a non-zero δ_{VO} develops (PBE+U+J predicts a smaller critical U_{V} than LDA+U+J). This is because a Hubbard U_{V} favors d orbital splitting (d_{xy} is split from d_{xz} and d_{yz}) and the resulting orbital ordering ($d^1_{xy}d^0_{xz}d^0_{yz}$) in $BaVO_3$ can stabilize a non-zero δ_{VO}. Since experimentally bulk perovskite $BaVO_3$ is cubic (i.e. $\delta_{\text{VO}} = 0$) and one of the key predictions of double perovskite Ba_2VFeO_6 is that a non-zero δ_{VO} develops after the charge transfer, we use $U_{\text{V}} = 3$ eV to calculate double perovskite oxides. This is to make sure that a non-zero δ_{VO} is not an artifact of a large value of Hubbard U_{V}. We note the value $U_{\text{V}} = 3$ eV is slightly smaller than that used on $SrVO_3$ in other works. We also re-perform all the calculations using $U_{\text{V}} = 5$ eV and all the results are qualitatively consistent with those calculated using $U_{\text{V}} = 3$ eV.

Bulk perovskite $BaFeO_3$ is a cubic ferromagnet. Its ground state has ferromagnetic ordering and a local magnetic moment of 3.5 μ_B per Fe site. We fully relax the atomic structure using a few different values of U_{Fe} and calculate the local magnetic moment on Fe in the ferromagnetic state. First we find that as U_{Fe} changes from 3 to 6 eV, no δ_{FeO} develops in either PBE+U+J or LDA+U+J. The ground state always has a cubic structure. Second, the values of local magnetic moment on Fe are shown in panels **b1** and **b2** of Fig. 2. The Fe-projected magnetic moment increases with U_{Fe} and $U_{\text{Fe}} = 5$ eV generates best agreement with the experiment values. Finally, we use $U_{\text{Fe}} = 5$ eV to calculate different magnetic ordering for $BaFeO_3$ and show the energy difference ΔE between different magnetic orderings in panels **c1** and **c2** of Fig. 2. The results show that at $U_{\text{Fe}} = 5$ eV, both PBE+U+J and LDA+U+J methods predict that ferromagentism is the most stable magnetic ordering, consistent with the experiment.

The density of states for cubic $BaVO_3$ and $BaFeO_3$ are shown in Fig. 3 and Fig. 4,

respectively. For $BaVO_3$, the ground state has A-type antiferromagnetic ordering. For $BaFeO_3$, the ground state has ferromagnetic ordering.

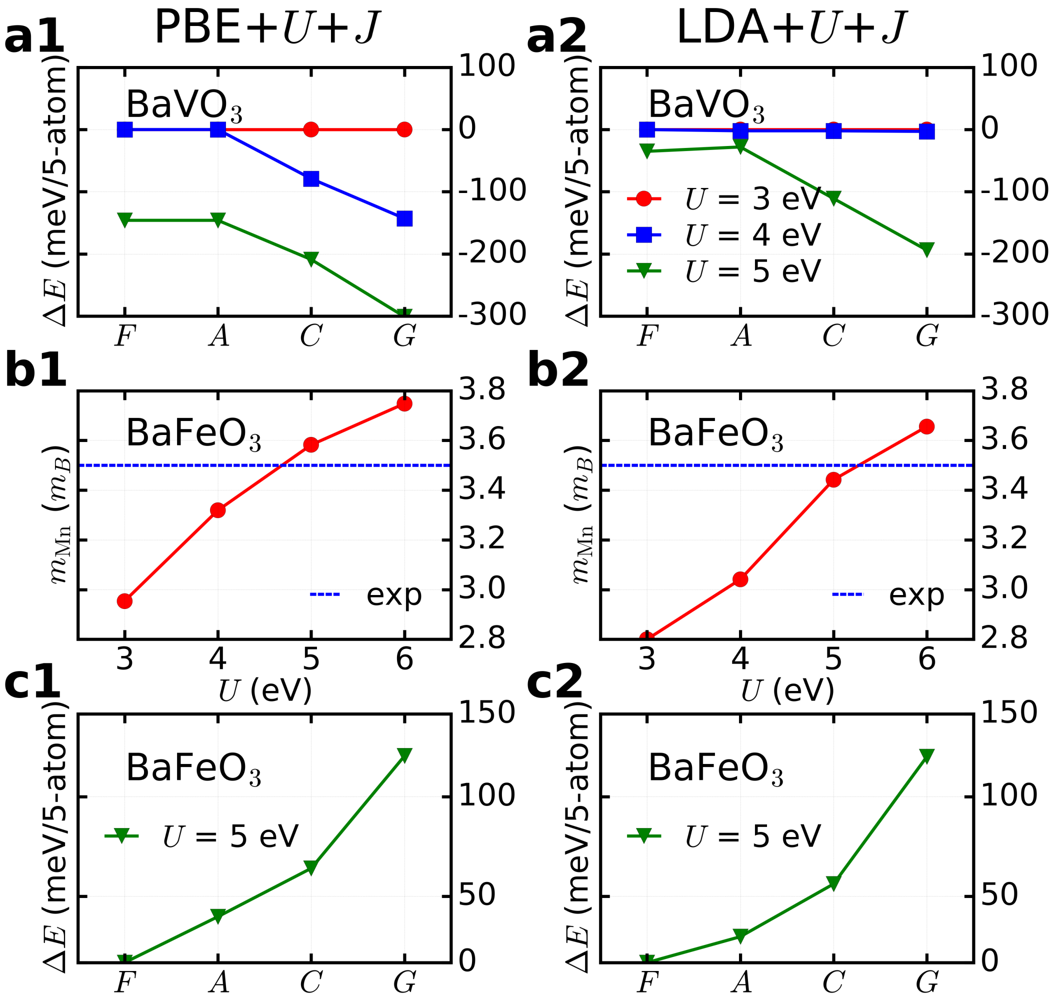

FIG. 2: **Bulk properties of perovskite $BaVO_3$ and $BaFeO_3$ caculated using DFT+U methods**. The left column (column **1**) is the results calculated using PBE+U+J method. The right column (column **2**) is the results calculated using LDA+U+J method. $J_{\rm V} = J_{\rm Fe} = 0.7$ eV is fixed in both methods. Panels **a1** and **a2** show the energy difference ΔE between the non-centrosymmetric structure and the cubic structure of perovskite $BaVO_3$. The unit is meV per 5-atom formula. F, A, C, G are different magnetic orderings. The red dots, blue squares and green triangles are the results for U = 3, 4 and 5 eV, respectively. Panels **b1** and **b2** show the Mn-projected magnetic moment of perovskite $BaFeO_3$ with the ferromagnetic ordering. The blue dashed line is the experimental value. Panels **c1** and **c2** show the energy difference between different magnetic orderings of perovskite $BaFeO_3$, calculated using $U_{\rm Fe}$ = 5 eV. The reference is the ferromagnetic state (F) and the unit is meV per 5-atom formula.

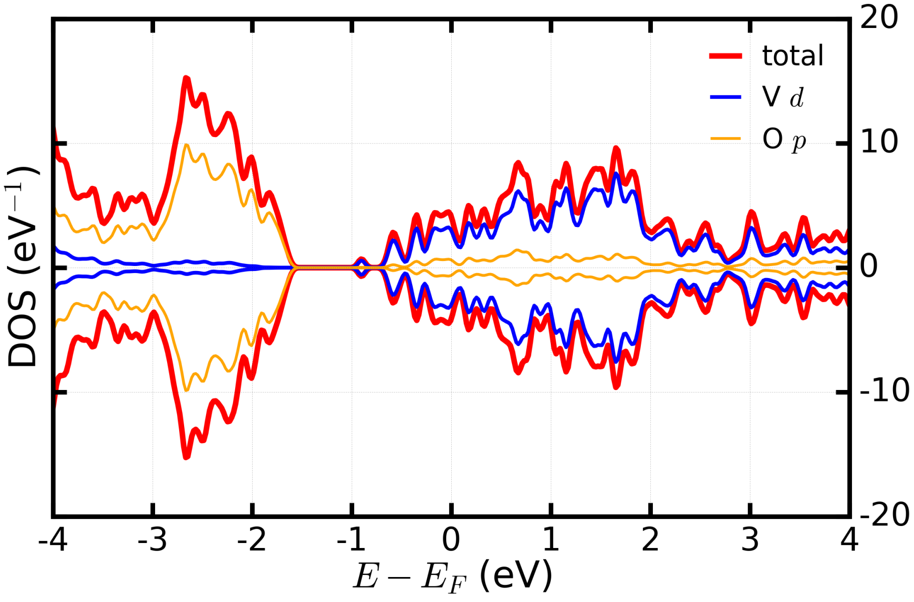

FIG. 3: Density of states of cubic $BaVO_3$ with A-type antiferromagnetic ordering, calculated using PBE+U+J with $U_V = 3$ eV and $J_V = 0.7$ eV.

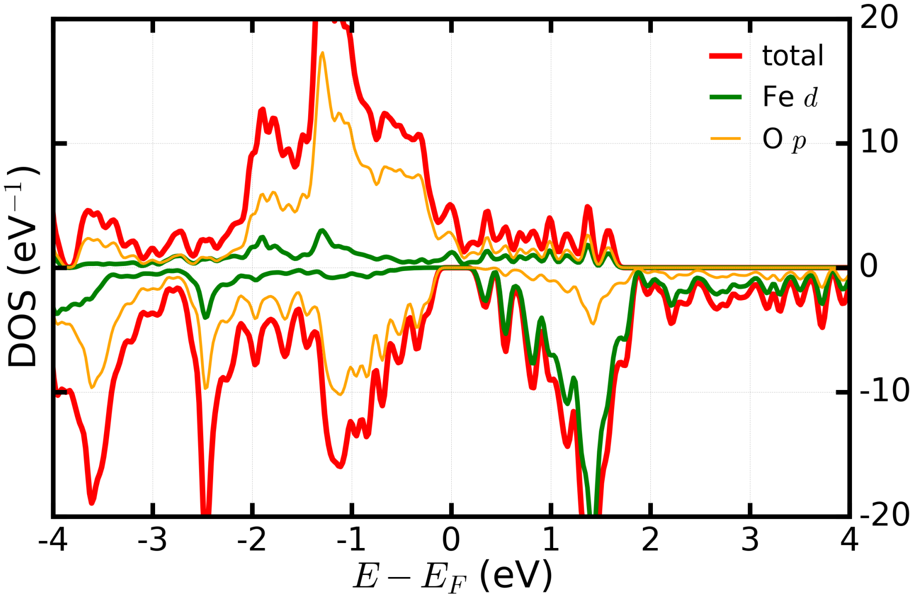

FIG. 4: Density of states of cubic $BaFeO_3$ with ferromagnetic ordering, calculated using PBE+U+J with $U_{Fe} = 5$ eV and $J_{Fe} = 0.7$ eV.

III. DETERMINATION OF OPTICAL TRANSITION MATRIX AND OPTICAL GAP

Optical conductivities are calculated via complex dielectric constant:

$$\epsilon(\omega) = \epsilon_0 + \frac{i\sigma(\omega)}{\omega} \tag{1}$$

Dielectric constants are calculated via summing interband transitions:

$$\mathrm{Im}\epsilon_{\alpha\beta}(\omega) = \frac{4\pi^2 e^2}{\Omega} \lim_{q\to 0} \frac{1}{q^2} \sum_{c,v,\mathbf{k}} 2\omega_{\mathbf{k}} \delta(\epsilon_{c\mathbf{k}} - \epsilon_{v\mathbf{k}} - \omega) \times \langle u_{c\mathbf{k}+\mathbf{e}_\alpha q} | u_{v\mathbf{k}} \rangle \langle u_{c\mathbf{k}+\mathbf{e}_\beta q} | u_{v\mathbf{k}} \rangle^* \tag{2}$$

where c, v refer to conduction and valence bands. α, β refer to x, y, z directions. $\mathbf{e}_\alpha$ is a unit vector along the direction of α. The real part of dielectric constant $\mathrm{Re}\epsilon_{\alpha\beta}(\omega)$ is obtained via Kramers-Kronig transformation.

Double perovskite Ba_2VFeO_6 has a direct gap, but its optical gap is larger than the direct gap. Fig. 5 shows the contribution to optical conductivity only from the Γ point, i.e. the summation in Eq. (2) only takes into account the contribution of $\mathbf{k} = \Gamma$. We can see that the first peak at $\omega = 1.10$ eV corresponds to the minimal optical excitation, while the fundamental gap is at $\omega = 0.79$ eV, highlighted by the green arrow.

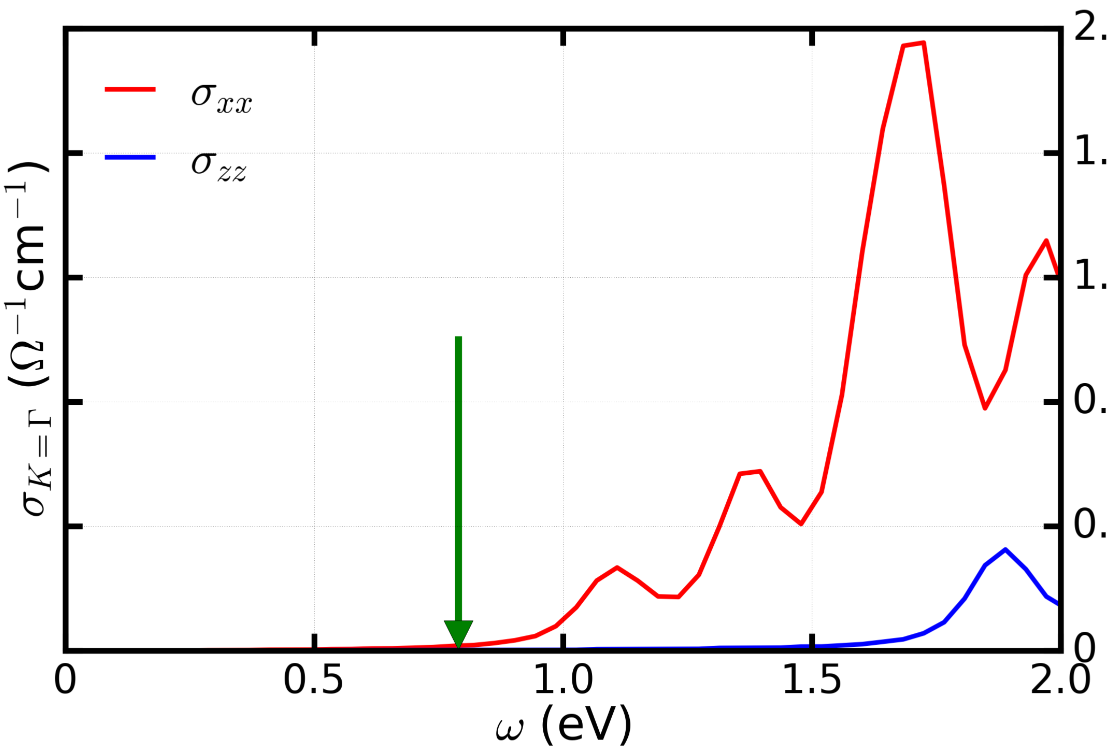

FIG. 5: Γ point optical conductivity σ of tetragonal Ba_2VFeO_6. The red lines are for the xx-component and the blue lines are for the zz-component. The green arrows indicate the fundamental gap of band structures.

IV. CRYSTAL STRUCTURES OF BA_2VFEO_6

We provide the crystal structure of Ba_2VFeO_6 in both cubic (space group Fm-$3m$) and tetragonal structures (space group $I4/m$).

TABLE I: Extension of Table I in the main text. Crystal structure of cubic Ba_2VFeO_6 (space group Fm-$3m$). x, y, z are fractional coordinates. a is the lattice constant.

	PBE+U+J			LDA+U+J			sPBEsol		
a (Å)	4.016			3.922			3.965		
	x	y	z	x	y	z	x	y	z
Ba1	0	0	0	0	0	0	0	0	0
Ba2	0.5	0	0	0.5	0	0	0.5	0	0
Ba3	0	0.5	0	0	0.5	0	0	0.5	0
Ba4	0.5	0.5	0	0.5	0.5	0	0.5	0.5	0
Ba5	0	0	0.5	0	0	0.5	0	0	0.5
Ba6	0.5	0	0.5	0.5	0	0.5	0.5	0	0.5
Ba7	0	0.5	0.5	0	0.5	0.5	0	0.5	0.5
Ba8	0.5	0.5	0.5	0.5	0.5	0.5	0.5	0.5	0.5
V1	0.25	0.25	0.25	0.25	0.25	0.25	0.25	0.25	0.25
V2	0.75	0.75	0.25	0.75	0.75	0.25	0.75	0.75	0.25
V3	0.75	0.25	0.75	0.75	0.25	0.75	0.75	0.25	0.75
V4	0.25	0.75	0.75	0.25	0.75	0.75	0.25	0.75	0.75
Fe1	0.25	0.75	0.25	0.25	0.75	0.25	0.25	0.75	0.25
Fe2	0.75	0.25	0.25	0.75	0.25	0.25	0.75	0.25	0.25
Fe3	0.25	0.25	0.75	0.25	0.25	0.75	0.25	0.25	0.75
Fe4	0.75	0.75	0.75	0.75	0.75	0.75	0.75	0.75	0.75
O1	0.25	0.009	0.25	0.25	0.008	0.25	0.25	0.009	0.25
O2	0.009	0.25	0.25	0.008	0.25	0.25	0.009	0.25	0.25
O3	0.75	0.991	0.25	0.75	0.992	0.25	0.75	0.991	0.25
O4	0.991	0.75	0.25	0.992	0.75	0.25	0.991	0.75	0.25
O5	0.491	0.25	0.25	0.492	0.25	0.25	0.491	0.25	0.25
O6	0.25	0.491	0.25	0.25	0.492	0.25	0.25	0.491	0.25
O7	0.75	0.509	0.25	0.75	0.508	0.25	0.75	0.509	0.25
O8	0.509	0.75	0.25	0.508	0.75	0.25	0.509	0.75	0.25
O9	0.25	0.25	0.0095	0.25	0.25	0.008	0.25	0.25	0.009
O10	0.75	0.25	0.9905	0.75	0.25	0.992	0.75	0.25	0.991
O11	0.25	0.75	0.9905	0.25	0.75	0.992	0.25	0.75	0.991
O12	0.75	0.75	0.0095	0.75	0.75	0.008	0.75	0.75	0.009
O13	0.25	0.991	0.75	0.25	0.992	0.75	0.25	0.991	0.75
O14	0.991	0.25	0.75	0.992	0.25	0.75	0.991	0.25	0.75
O15	0.75	0.009	0.75	0.75	0.008	0.75	0.75	0.009	0.75
O16	0.009	0.75	0.75	0.008	0.75	0.75	0.009	0.75	0.75
O17	0.509	0.25	0.75	0.508	0.25	0.75	0.509	0.25	0.75
O18	0.25	0.509	0.75	0.25	0.508	0.75	0.25	0.509	0.75
O19	0.491	0.75	0.75	0.492	0.75	0.75	0.491	0.75	0.75
O20	0.75	0.491	0.75	0.75	0.492	0.75	0.75	0.491	0.75
O21	0.25	0.25	0.4905	0.25	0.25	0.492	0.25	0.25	0.491
O22	0.75	0.25	0.5095	0.75	0.25	0.508	0.75	0.25	0.509
O23	0.25	0.75	0.5095	0.25	0.75	0.508	0.25	0.75	0.509
O24	0.75	0.75	0.4905	0.75	0.75	0.492	0.75	0.75	0.491

TABLE II: Extension of Table I in the main text. Crystal structure of tetragonal Ba_2VFeO_6 (space group $I4/m$). x, y, z are fractional coordinates. a is the in-plane lattice constant and c is the out-of-plane lattice constant.

	PBE+U+J			LDA+U+J			sPBEsol		
a (Å)	3.958			3.916			3.946		
c (Å)	4.267			3.943			4.041		
	x	y	z	x	y	z	x	y	z
Ba1	-0.00000	-0.00000	0.02480	-0.00000	-0.00000	0.01695	-0.00000	-0.00000	0.01894
Ba2	0.50000	0.00000	0.02480	0.50000 -	0.00000	0.01695	0.50000	0.00000	0.01894
Ba3	-0.00000	0.50000	0.02480	-0.00000	0.50000	0.01695	0.00000	0.50000	0.01894
Ba4	0.50000	0.50000	0.02480	0.50000	0.50000	0.01695	0.50000	0.50000	0.01894
Ba5	-0.00000	0.00000	0.52480	0.00000	-0.00000	0.51695	0.00000	-0.00000	0.51894
Ba6	0.50000	-0.00000	0.52480	0.50000	-0.00000	0.51695	0.50000	0.00000	0.51894
Ba7	-0.00000	0.50000	0.52480	-0.00000	0.50000	0.51695	0.00000	0.50000	0.51894
Ba8	0.50000	0.50000	0.52480	0.50000	0.50000	0.51695	0.50000	0.50000	0.51894
V1	0.25000	0.25000	0.28105	0.25000	0.25000	0.27093	0.25000	0.25000	0.27514
V2	0.75000	0.75000	0.28105	0.75000	0.75000	0.27093	0.75000	0.75000	0.27514
V3	0.75000	0.25000	0.78105	0.75000	0.25000	0.77093	0.75000	0.25000	0.77514
V4	0.25000	0.75000	0.78105	0.25000	0.75000	0.77093	0.25000	0.75000	0.77514
Fe1	0.25000	0.75000	0.28934	0.25000	0.75000	0.27334	0.25000	0.75000	0.27958
Fe2	0.75000	0.25000	0.28934	0.75000	0.25000	0.27334	0.75000	0.25000	0.27958
Fe3	0.25000	0.25000	0.78934	0.25000	0.25000	0.77334	0.25000	0.25000	0.77958
Fe4	0.75000	0.75000	0.78934	0.75000	0.75000	0.77334	0.75000	0.75000	0.77958
O1	0.25000	0.00942	0.25825	0.25000	0.00834	0.26243	0.25000	0.00897	0.26080
O2	0.00942	0.25000	0.25825	0.00834	0.25000	0.26243	0.00897	0.25000	0.26080
O3	0.75000	0.99058	0.25825	0.75000	0.99166	0.26243	0.75000	0.99103	0.26080
O4	0.99058	0.75000	0.25825	0.99166	0.75000	0.26243	0.99103	0.75000	0.26080
O5	0.49058	0.25000	0.25825	0.49166	0.25000	0.26243	0.49103	0.25000	0.26080
O6	0.25000	0.49058	0.25825	0.25000	0.49166	0.26243	0.25000	0.49103	0.26080
O7	0.75000	0.50942	0.25825	0.75000	0.50834	0.26243	0.75000	0.50897	0.26080
O8	0.50942	0.75000	0.25825	0.50834	0.75000	0.26243	0.50897	0.75000	0.26080
O9	0.25000	0.25000	0.00895	0.25000	0.25000	0.01900	0.25000	0.25000	0.01583
O10	0.75000	0.25000	0.98804	0.75000	0.25000	0.00310	0.75000	0.25000	0.99837
O11	0.25000	0.75000	0.98804	0.25000	0.75000	0.00310	0.25000	0.75000	0.99837
O12	0.75000	0.75000	0.00895	0.75000	0.75000	0.01900	0.75000	0.75000	0.01583
O13	0.25000	0.99058	0.75825	0.25000	0.99166	0.76243	0.25000	0.99103	0.76080
O14	0.99058	0.25000	0.75825	0.99166	0.25000	0.76243	0.99103	0.25000	0.76080
O15	0.75000	0.00942	0.75825	0.75000	0.00834	0.76243	0.75000	0.00897	0.76080
O16	0.00942	0.75000	0.75825	0.00834	0.75000	0.76243	0.00897	0.75000	0.76080
O17	0.50942	0.25000	0.75825	0.50834	0.25000	0.76243	0.50897	0.25000	0.76080
O18	0.25000	0.50942	0.75825	0.25000	0.50834	0.76243	0.25000	0.50897	0.76080
O19	0.49058	0.75000	0.75825	0.49166	0.75000	0.76243	0.49103	0.75000	0.76080
O20	0.75000	0.49058	0.75825	0.75000	0.49166	0.76243	0.75000	0.49103	0.76080
O21	0.25000	0.25000	0.48804	0.25000	0.25000	0.50310	0.25000	0.25000	0.49837
O22	0.75000	0.25000	0.50895	0.75000	0.25000	0.51900	0.75000	0.25000	0.51583
O23	0.25000	0.75000	0.50895	0.25000	0.75000	0.51900	0.25000	0.75000	0.51583
O24	0.75000	0.75000	0.48804	0.75000	0.75000	0.50310	0.75000	0.75000	0.49837
